# Supplementary material for: DNA Barcoding and ITS2 Secondary Structure Predictions in Taro (Colocasia esculenta L. Schott) from the North Eastern Hill Region of India
Source: Genes (Basel). 2022 Dec 5;13(12):2294. doi: 10.3390/genes13122294 (PMC9778394; doi:10.3390/genes13122294)
Supplement: Supplementary file 1 [file genes-13-02294-s001.zip › genes-2001301-supplementary.pdf]

Table S1. List of the twenty taro genotypes used in the study.

Voucher ID	Genotypes	Place of Collection	Geographical location
RC MNP T1	RCMC -1	ICAR RC NEHR, Manipur	24° 50′ N, 93° 55′ E
RC MNP T2	RCMC -2	ICAR RC NEHR, Manipur	24° 50′ N, 93° 55′ E
RC MNP T3	RCMC -5	ICAR RC NEHR, Manipur	24° 50′ N, 93° 55′ E
RC MNP T4	RCMC -6	ICAR RC NEHR, Manipur	24° 50′ N, 93° 55′ E
RC MNP T5	RCMC -10	ICAR RC NEHR, Manipur	24° 50′ N, 93° 55′ E
RC MNP T6	DP-25	RC CTCRI, Bhubaneswar	20° 14′ N, 85° 47′ E
RC MNP T7	Duradim	Umroi Road, Meghalaya	25° 40′ N, 91° 55′ E
RC MNP T8	Jhankri	RC CTCRI, Bhubaneswar	20° 14′ N, 85° 47′ E
RC MNP T9	Muktakeshi (MK)	RC CTCRI, Bhubaneswar	20° 14′ N, 85° 47′ E
RC MNP T10	Topi	RC CTCRI, Bhubaneswar	20° 14′ N, 85° 47′ E
RC MNP T11	Satasankha	RC CTCRI, Bhubaneswar	20° 14′ N, 85° 47′ E
RC MNP T12	TSL	RC CTCRI, Bhubaneswar	20° 14′ N, 85° 47′ E
RC MNP T13	P Chandel	Chandel, Manipur	24° 19′ N, 94° 01′ E
RC MNP T14	R5JH10	ICAR RC NEHR, Manipur	24° 50′ N, 93° 55′ E
RC MNP T15	R5MK	ICAR RC NEHR, Manipur	24° 50′ N, 93° 55′ E
RC MNP T16	R1B9	ICAR RC NEHR, Manipur	24° 50′ N, 93° 55′ E
RC MNP T17	PCT1	ICAR RC NEHR, Manipur	24° 50′ N, 93° 55′ E
RC MNP T18	BBSL	RC CTCRI, Bhubaneswar	20° 14′ N, 85° 47′ E
RC MNP T19	TOPI-1kR	ICAR RC NEHR, Manipur	24° 50′ N, 93° 55′ E
RC MNP T20	BBSR	RC CTCRI, Bhubaneswar	20° 14′ N, 85° 47′ E
